# Revealing Individual Signatures of Human T Cell CDR3 Sequence Repertoires with Kidera Factors

**DOI:** 10.1371/journal.pone.0086986

**Published:** 2014-01-29

**Authors:** Michael Epstein, Martino Barenco, Nigel Klein, Michael Hubank, Robin E. Callard

**Affiliations:** 1 Department of Statistical Science, University College London, London, United Kingdom; 2 CoMPLEX, University College London, London, United Kingdom; 3 Institute of Child Health, University College London, London, United Kingdom; Chang Gung University, Taiwan

## Abstract

The recent development of High Throughput Sequencing technologies has enabled an individual’s TCR repertoire to be efficiently analysed at the nucleotide level. However, with unique clonotypes ranging in the tens of millions per individual, this approach gives a surfeit of information that is difficult to analyse and interpret in a biological context and gives little information about TCR structural diversity. Using publicly available TCR CDR3 sequence data, we analysed TCR repertoires by converting the encoded CDR3 amino acid sequences into Kidera Factors, a set of orthogonal physico-chemical properties that reflect protein structure. This approach enabled the TCR repertoire from different individuals to be distinguished and demonstrated the close similarity of the repertoire in different samples from the same individual.

## Introduction

It has been estimated that potentially 4.2×10^17^ different α-β T-cell receptors (TCR) can be generated during maturation in the thymus [1]. This diversity stems from the combinatorial rearrangement of germline DNA during thymocyte differentiation. Both chains of the receptor are encoded by a number of genes (V, J and C for the alpha chain and V, D, J and C in the beta chain) from which only a subset is selected to form the TCR. For example, the human alpha chain can be constructed from a choice of 94–96 functional components [2], of which approximately 43–45 are Variable genes; 50 are Joining genes and one is a Constant gene segment, leading to around 2,200 possible V-J-C combinations. Further diversity is provided through the CDR3 region situated between the V and J segments within each of these chains by addition, deletion and substitution of bases at the 3′ end of the variable segment and the 5′ end of the joining segment. Combination of the rearranged alpha chain with a similarly rearranged β-chain incorporating additional variation provided by the D region, accounts for the enormous overall diversity in T-cell receptors. This diversity of CDR3 regions is crucial in providing an appropriate response to a broad range of pathogens. Knowledge of the CDR3 sequence content in an individual is therefore of central importance in immunology [3]–[6].

CDR3 diversity has been estimated previously by spectratyping, whereby the length distribution of the CDR3 region in a T-cell receptor is obtained by gel electrophoresis [7], [8]. This method is cheap and fast and has been used in numerous studies using signature bias of CDR3 regions to detect and describe the impact of pathologies such as HIV [9] and Multiple Sclerosis [10]. The drawback of spectratyping is that it ignores the actual sequence content of the CDR3 regions. Subsequent methods have employed traditional Sanger sequencing of T-cell receptor PCR products of small numbers of T-cells. This has the merit of assessing CDR3 sequence identity [11] but the sequencing cost per base and constraints on the number of cells that can reasonably be analysed [12] makes the technique impracticable for assessing CDR3 diversity in individual blood samples. More recently, High Throughput Sequencing (HTS) technologies have enabled millions of TCR clonotypes (the receptor transcript which comprises the V,(D),J,C gene usage including the CDR3 region to be identified [5], [13], [14]. HTS comes with its own challenges, however, including sequencing errors, priming and PCR biases. For example, the error rate is rather higher with HTS compared to Sanger sequencing (0.1–1% vs. 0.01–0.001%, depending on the sequencing technology [12]). There is a risk therefore of measuring artefactual diversity, originating from the measurement apparatus rather than from the underlying true sequences. To overcome these issues, conservative quality control methods have been designed and thoroughly tested using real and artificially generated data [14]–[16]. A second problem related to the use of HTS techniques stems paradoxically from the very success of the technology itself. From the relative data paucity of the recent past, there is now a potential information overload that can be difficult to interpret. As an example, a total of 1,061,552 different clonotypes from peripheral blood samples of healthy individuals have recently been described [14]. Direct comparison of the TCR sequences in samples from different individuals and between samples from one individual found few intersecting nucleotide sequences within libraries [14]. Indeed, when sampling the *same* individual a week later, only 12.8% of nucleotide sequences intersected within the two samples indicating that only a fraction of the true sequence diversity appears to be captured in a single blood sample. It is difficult therefore to attribute a sample to an individual on the basis of direct comparisons of individual sequences. In addition, such “Venn-based” indicators are likely to depend heavily on the size of the sample and are therefore difficult to interpret.

Here, we address the challenge of interpreting information contained in clonotype sequences produced by HTS [17] by transforming CDR3 sequences into a set of physico-chemical properties of the encoded amino acids (Kidera Factors) [18], which are known to be related to 3D protein structure [19]. Using statistical approaches borrowed from ecology, we then examine, and explicitly compare TCR receptor diversity within and between individuals. Our results show that TCR diversity between individuals but not within individuals can be distinguished by this approach.

## Methods

### TCR Receptor CDR3 Sequences

Files containing all the distinct TCRβ and corresponding amino acid sequences used in this study are hosted online at ftp://ftp.bcgsc.ca/supplementary/TCRb2010/ [14]. Sequences from eight blood samples from 3 individuals (two males and one female unrelated to each other) were studied in detail (Table 1). Six samples of unseparated, naïve and memory T-cells taken twice one week apart were all from one donor (Male 1 in Table 1). The other samples of unseparated T cells were derived from single blood draws from the other male (Male 2) and a female (Female). The number of unique clonotypes (V-CDR3-J ) and CDR3 sequences identified in each of these eight samples is also given in Table 1.

**Table 1:** Blood samples and associated clonotypes of publically available TCRβ sequences.

T cell preparation	Sample*	Unique TCRβ ntsequences (V-CDR3-J)	Unique CDR3 aminoacid sequences	Unique KideraClonotypes
M1 unseparated	1	494,796	419,990	337,745
M1 unseparated	2	352,139	312,445	260,732
M1 memory	1	52,166	50,748	47,955
M1 memory	2	83,206	75,920	70,178
M1 naïve	1	55,253	52,137	49,130
M1 naïve	2	121,233	102,788	95,042
M2 unseparated	1	193,551	165,931	146,445
F1 unseparated	1	93,990	86,225	78,391

Male 1 (M1), Male 2 (M2) and Female 1 (F1).

* Unseparated, memory and naïve samples 1 and 2 were taken one week apart.

Descriptive statistics of the eight samples generated by [14]. This includes the number of unique clonotypes (V-CDR3-J rearrangements) and the number of unique CDR3 amino acid sequences ignoring the Variable and Joining gene usage. The number of unique Kidera Factor clonotypes defined by the 10 dimensional vector is compared to the number of unique CDR3 amino acid sequences derived from the nucleotide (nt) sequences described by Warren et al [14].

### Generating Kidera Factor Representations of Clonotype Sequences

The Kidera Factors were originally derived by applying multivariate analysis to 188 physical properties of the 20 amino acids and using dimension reduction techniques [18]. A 10-dimensional vector of orthogonal factors was then obtained for each amino acid (an example representation for Alanine is given in supplementary material Table S1) that explained 86% of the variance in the original dataset. A connection between the Kidera Factors and the structural properties of proteins was established by encoding a set of proteins from the CATH database using Kidera Factors and then applying Principal Component Analysis (PCA) to demonstrate relationships between proteins that have similar structures but unknown sequence homology [19], [20]. This approach was able to identify remote protein homologues missed by conventional homology modelling.

The CDR3 region within each clonotype is defined as the sequence between the conserved Cys amino acid of the Variable gene and the conserved Phe amino acid of the Joining gene. Ten-dimensional Kidera Factor representations [18] (Table S1) for each CDR3 sequence were computed by taking the average score for each Kidera factor across all amino acids within the CDR3 sequence. This resulted in an average score for each of the Kidera Factors represented as a ten dimensional vector and inserted into a csv file. The raw sql files from [14] were concatenated with the Kidera Factor representation for each clonotype, converted into csv format and imported into R for data analysis.

### Principal Component Analyses and Multidimensional Scaling

**Figure 1:**
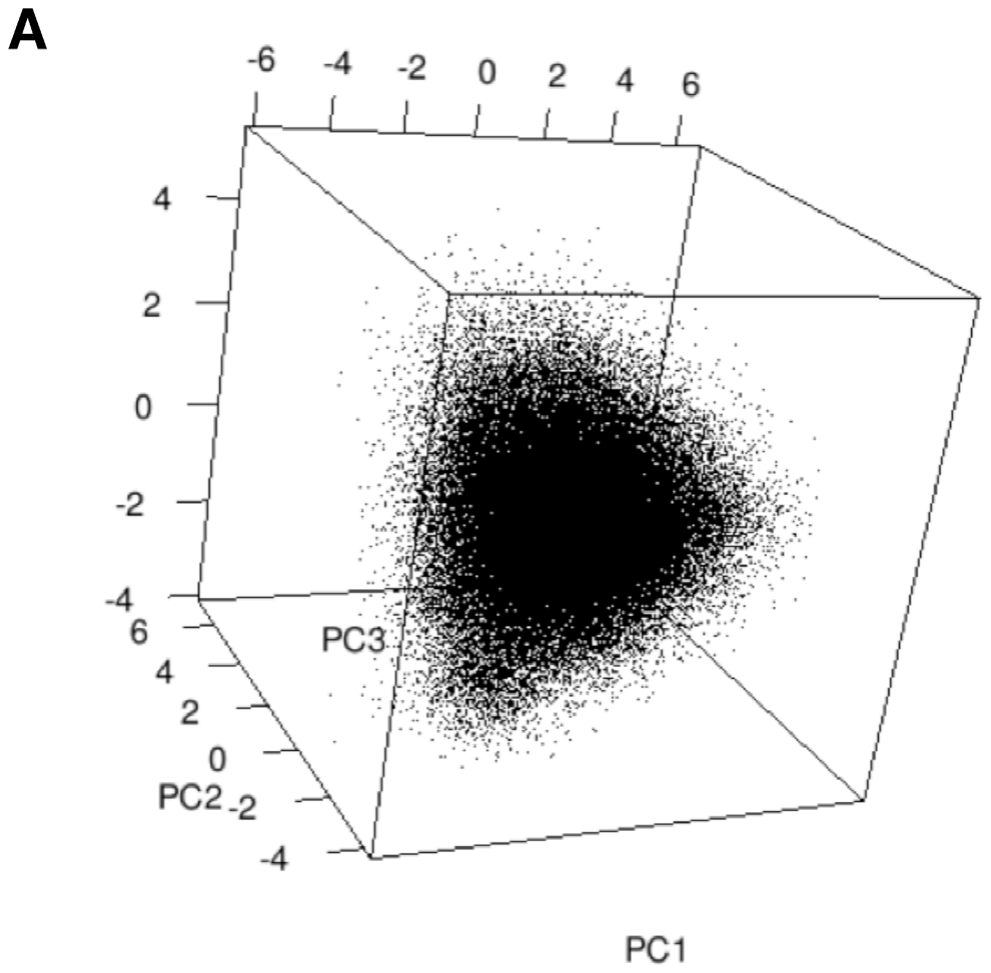
PCA of Kidera Factor representation of unique CDR3 sequences. 3D scatterplot on the first three eigenvectors of the Principal Component Space obtained from Kidera Factor representations of the unique CDR3 sequences in the unseparated T cell Female sample. In this sample there are 78,391 unique Kidera Factor representations of 86,225 unique CDR3 sequences (Table 1).

Kidera Factor vectors of the CDR3 regions were subject to PCA and plotted using the rgl and ggplot2 packages in R. Generating a Kidera Factor representation for each CDR3 region in all of the clonotypes from the samples shown in Table 1 resulted in hundreds of thousands of transformed sequences (Kidera Factor vectors, Figure 1). To analyse these data further, the Kidera Factor representations for each CDR3 region were aggregated with the Variable genes contained within that sample. This produced the average physico-chemical properties of the CDR3 region per Variable gene in each sample and enabled a significant reduction in the dimensionality of the data. Multi-dimensional scaling was then employed to preserve the relative similarity between Variable gene aggregates of different samples, in a lower dimension, using Euclidean distance between the Kidera Factor vectors as the similarity measure. This helped explore intuitively whether the Kidera Factor repertoires in the different samples were distinguishable. In the course of MDS fitting, two samples were found to have a Vβ gene (TRBV-17) with the same CDR3 Kidera Factor aggregation leading to a Euclidean distance of 0 between these two points. In these instances, a small delta (0.000000001) was applied to allow the non-metric MDS algorithm to complete.

### Computation of the ANOSIM Indicators and P-values

The aim of the non-parametric ANOSIM (Analysis of Similarity) test is to judge whether two clonotype samples can be distinguished statistically. The procedure tests whether the CDR3 Kidera Factors from one sample are more similar to each other than to those from a different sample. If this is the case, the samples are considered to be distinguishable from each other. The test is computed on the rank order of similarities between all CDR3 Kidera Factor vectors within the two samples derived by comparing the Euclidean distance between the 10 dimensional Kidera representations of the two chosen samples. The closer the Euclidean distance between two Kidera Factor vectors, the more similar they are considered to be.

Following the convention described in [21], the matrix of rank similarities was sub-partitioned by sample (See Supplementary Material Figure S1 for an example calculation). *r_B_* is denoted to be the average rank similarity between samples and *r_w_* to be the average rank within each sample. The test statistic is constructed as:
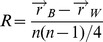



A small or negative R-statistic indicates no difference between the rank similarity between the groups. In contrast, if the samples are well separated *r_B_* will exceed *r_W_* and the R-statistic will be positive. The theoretical range of the R-statistic is between −1 and 1. In practice this range is much smaller and as in any statistical test, required a comparison of the R-statistic with a null distribution to derive a p-value (see Figure S1).

### Programming Code

All statistical analysis was performed using R (version 2.13.0) with external packages MASS (for non-metric MDS), ggplot2, scatterplot3d, rgl, mgcv and lattice for data visualisation. The ANOSIM test and null distribution generation was coded by one of us (ME) following the approach described by Clarke [21].

## Results

### PCA of Kidera Factor Vectors

**Figure 2:**
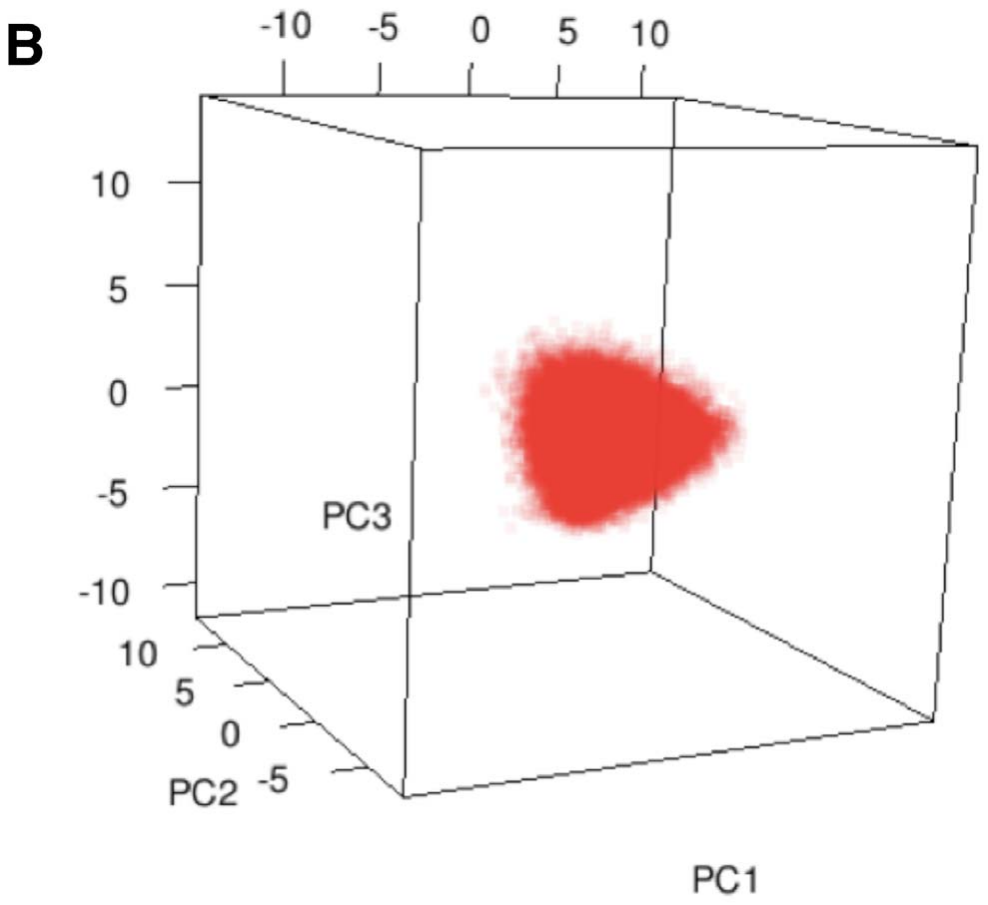
Diversity of Vβ CDR3 Kidera Factors. The full set of CDR3 Kidera Factors from an unseparated T cell sample was contained in a small fraction of the theoretical total possible diversity bounded by the maximum possible Kidera Factor values for each PCA. The example shown is from the Female unseparated sample which contained 78,391 unique Kidera Factors.

An average value for each of the ten Kidera Factors over each amino acid in the CDR3 sequences found in the clonotype dataset described by Warren et al [14] was calculated as described Rackovsky [19]. This resulted in an averaged ten-dimensional Kidera Factor vector for each CDR3 sequence. Notably, there were fewer unique Kidera factors in each sample than unique CDR3 amino acid sequences indicating less diversity than suggested by the CDR3 amino acid sequence data (Table 1). The full set of CDR3 Kidera Factor vectors was contained in a small fraction of the theoretical total possible diversity (Figure 2).

**Figure 3:**
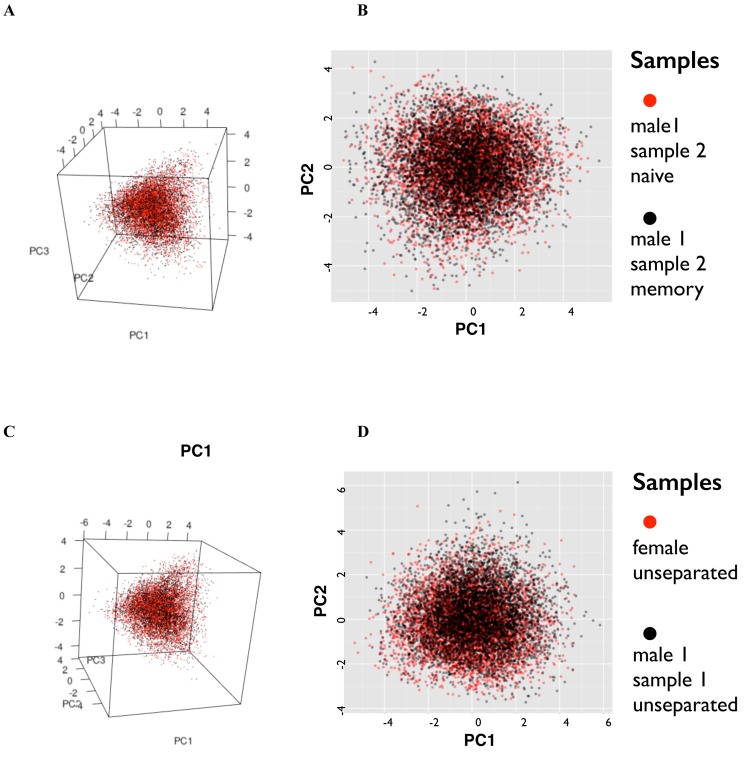
PCA of naïve and memory T cell samples from the same donor and unseparated T cell samples from different donors. The first three principal components of the naïve and memory samples from Male 1 sample 2 (A) and the first two principal components of the same analysis (B). Unseparated samples from Male 1 (sample 1) and Female on the first three principal components (C) and the first two principal component axes (D). The slight shift of the male points compared to the female points suggests that these samples contain different physicochemical properties (sample size = 5000).

Direct comparisons of the CDR3 Kidera Factor repertoires present in the various samples from different individuals were performed using Principal Component Analysis (PCA). The results for naïve and memory T cell subsets from the same individual (male1) and for unseparated cells from two different donors (male 1and female) are shown in Figure 3. Whereas the CDR3 DNA sequences of the naïve and memory cells were largely distinct with an overlap of only about 1% [14], 3D PCA analysis of the Kidera Factors showed a similar distribution of the two populations consistent with the memory T cells being a random sample of the naïve population (Figure 3A). Projection of 5,000 randomly selected datapoints from the naïve and memory samples on to a 2D plot also appears to indicate a similar distribution (Figure 3B).

Direct comparisons of the unique CDR3 Kidera Factors in unseparated T cell samples from different individuals performed using Principal Component Analysis suggested that the distributions were a little different (Figure 3C). This is most evident in the 2D PCA projection of 5,000 randomly selected samples in Figure 3D. The distribution of black points pertaining to the male sample were shifted away slightly from the red points of the female sample. Although this by itself cannot be considered conclusive evidence that the samples are physiochemically different, it suggested that further statistical analysis was warranted.

### Data Compression and Graphical Representation using Multidimensional Scaling (MDS)

Although PCA seemed to indicate some differences between samples from different individuals, this method did not allow the way they were distinguishable to be quantified. An indication of diversity can be obtained by enumerating the number of unique Kidera Factors (Table 1) but PCA by itself is not a quantitative method that allows for testing whether the two samples are distinct. This difficulty in quantitative comparison of PCA of different samples prompted us to analyse the data further using multidimensional scaling (MDS). To do this, the information from the Kidera Factors was compressed by aggregating the Kidera Factors of all CDR3 sequences according to their associated Vβ gene [14]. This has the advantage of reducing the information for each sample to a set of about fifty 10-dimensional vectors per sample and making the computational requirements for Multidimensional Scaling (MDS) much more economical than for the non-aggregated representation of the sample.

**Figure 4:**
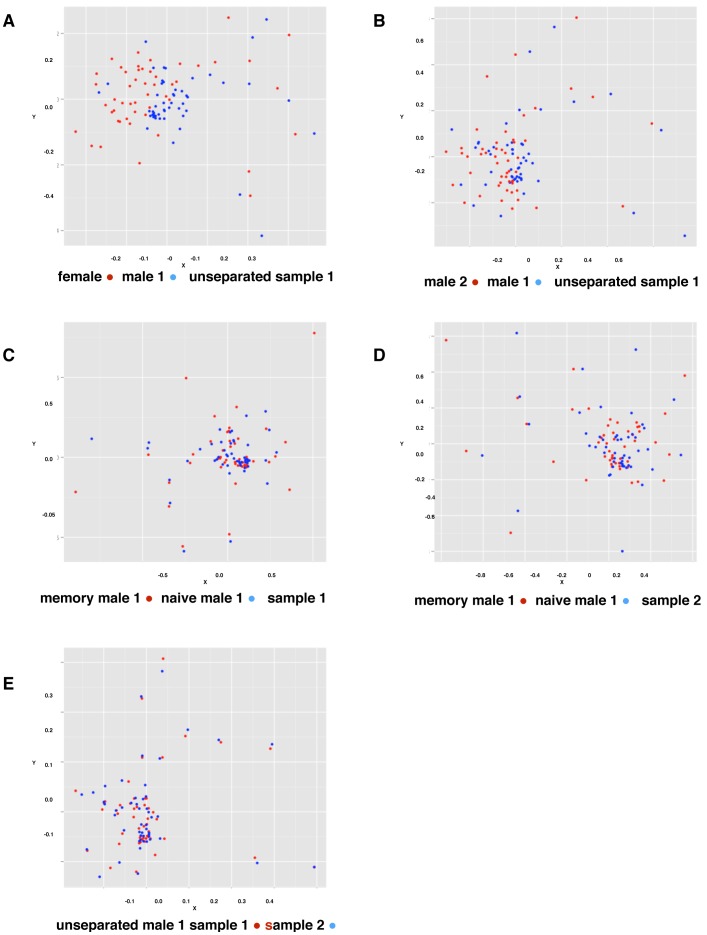
MDS representation of similarity between samples. Unseparated T cell samples from Female donor compared with Male 1, sample 1 (A) (2-D stress value 16.12%) and from Male 1 compared with Male 2 (B). Note the north-west south-east separation between the male and female samples and the two male samples. Naïve T cell sample compared with memory T cells from Male 1 (C) (2-D stress value 13.70%) and repeated one week later (D) (2-D stress value 14.52%); and unseparated T cell samples from Male 1 taken one week apart (E) (2-D stress value 11.72%).

MDS also provides a graphical visualisation of the data in a lower dimensional space, which preserves the relative similarity, reported in the output by Euclidian distance between individual data points. It does so by iteratively minimising the squared difference between the Euclidean distances proposed between the points in the reduced space and the actual rank dissimilarities between the points in the higher dimensional space. Two-dimensional MDS representations for unseparated T cells from different donors are presented in Figure 4A and 4B; naïve and memory T cells from the same donors in Figure 4C and 4D; and two unseparated samples from the same individual taken a week apart in Figure 4E. Although they are not entirely distinct, each sample from different donors appears to preferentially occupy different regions of the plane, broadly the lower right for male 1 and higher left for the female sample (Figure 4A) and lower left for the male 2 sample (Figure 4B). In contrast, no such distinction can be seen in the other panels depicting naïve and memory T cell populations from the same donor (Figure 4C and 4D) and unseparated samples drawn from the same individual, taken a week apart (Figure 4E). These results are consistent with the PCA results and indicate a difference in the CDR3 Kidera factor distribution between different individuals but not between different samples taken at different times from the same individual, or naïve and memory cells from the same individual.

### The ANOSIM Statistic for Testing Pair-wise Sample Combinations

**Figure 5:**
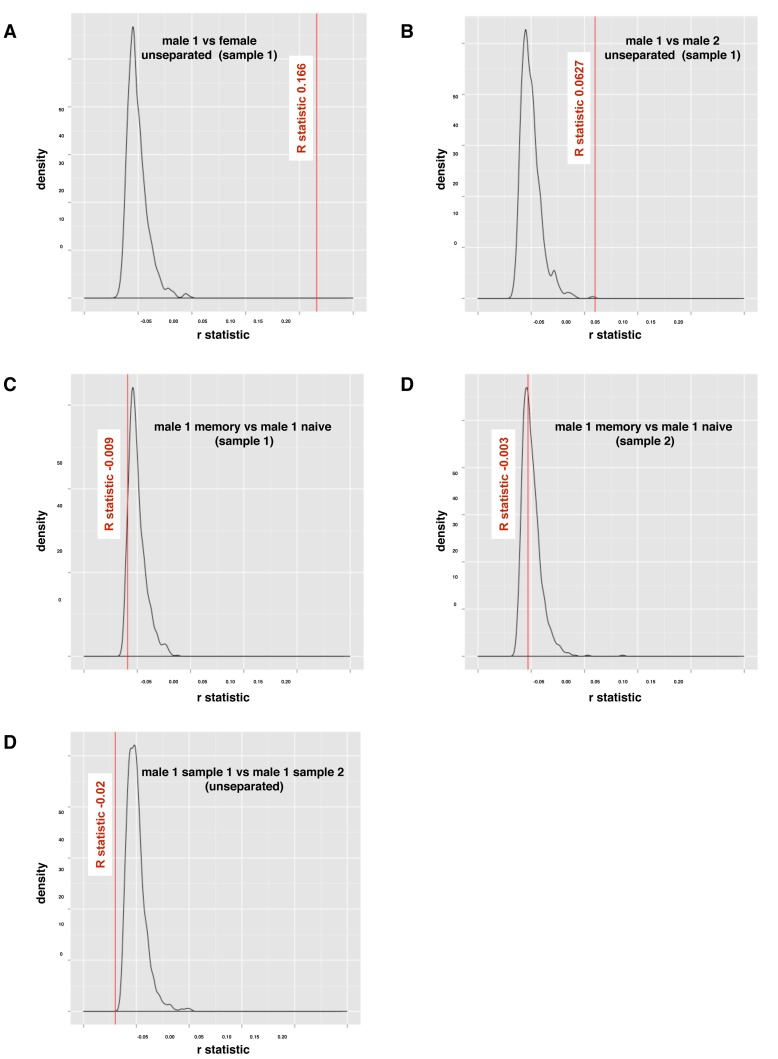
ANOSIM R-statistic and null distribution. ANOSIM statistic for unseparated T cell samples from Male 1 compared with Female, p<0.001 (A); Male 1 compared with Male 2, p ≈ 0.001 (B); Naïve T cell sample compared with memory T cells from Male 1, p≈1 (C); repeated one week later, p≈1 (D); and unseparated T cell samples from Male 1 taken one week apart, p≈1 (E).

Although visually the differences between the different samples seem clear enough, interpretation of graphic representations is still subjective. To overcome this, the non-parametric ANOSIM (Analysis of Similarity) test, commonly used in ecological statistics, was used to compare samples. This test relies entirely on quantitative considerations to establish the difference between population samples. Results are shown for unseparated samples from different individuals (Figure 5A and 5B), for naïve and memory cells from the same individual (Figure 5C and 5D) and for unseparated samples taken at different times from the same individual (Figure 5E). The value of the R-statistic when unseparated samples from different individuals (male 1 sample 1 and female, Table 1) are compared was 0.166 and for when male 1 sample 1 was compared with male 2 was 0.063, both of which significantly exceeded any values obtained through random reshuffles. Since a thousand such permutations were performed, the corresponding null hypothesis p-value does not exceed 0.001, thereby indicating that the two unseparated clonotype samples (male 1 sample 1 vs. female and male 1 sample 1 vs. male 2) are significantly different (P<0.001). In contrast, when naïve and memory cells from the same donor (Figure 5C and 5D) and two unseparated blood samples from the same individual (Figure 5E) are compared the R statistics are all slightly negative (−0.009, −0.004 and −0.02) with a large corresponding p-value of ≈ 1 showing that these samples are not statistically distinguishable from each other.

This analysis was repeated for all possible pairwise combinations of blood samples described in Table 1 and the results compiled in Table 2. The values of the R statistic are given below the diagonal whilst the corresponding p-values are given above the diagonal. It is clear that pairs of samples from different individuals (highlighted bold) are all significantly different (p<0.05). Conversely, for all pairwise comparisons belonging to the same individual, no significant differences were obtained (p>0.2). These results indicate that compressing the Kidera Factors data by aggregation with Vβ regions can successfully discriminate pairs of samples originating from different individuals, an outcome that is difficult to assess when the sequences are kept in their raw DNA sequence form and compared directly.

**Table 2:** ANOSIM R statistic (in italics below the diagonal) and p-values (above the diagonal) for all possible pairwise combinations of the eight human blood samples. Statistically different values highlighted in bold italic.

Donor	Male 1 S1Total T	Male 1 S2Total T	Male 1 S1memory	Male 1 S2memory	Male 1 S1naïve	Male 1 S2naïve	Male 2Total T	FemaleTotal T
**Male 1 S1 Total T**	xxx	1	0.548	0.891	1	0.642	**0.001**	**<0.001**
**Male 1 S2 Total T**	−*0.019*	xxx	0.604	0.961	1	0.55	**<0.001**	**<0.001**
**Male 1 S1 memory**	−*0.002*	−*0.004*	xxx	0.994	0.925	0.282	**<0.001**	**<0.001**
**Male 1 S2 memory**	−*0.009*	−*0.011*	−*0.013*	xxx	0.989	0.574	**<0.001**	**<0.001**
**Male 1 S1 naïve**	−*0.015*	−*0.016*	−*0.009*	−*0.013*	xxx	0.583	**<0.001**	**<0.001**
**Male 1 S2 naïve**	−*0.004*	−*0.002*	*0.003*	−*0.003*	−*0.003*	xxx	**<0.001**	**<0.001**
**Male 2 Total T**	*0.063*	*0.057*	*0.070*	*0.089*	*0.055*	*0.091*	xxx	**0.031**
**Female Total T**	*0.167*	*0.159*	*0.168*	*0.198*	*0.153*	*0.185*	*0.021*	xxx

This table shows the pairwise ANOSIM R-statistics (in italics below the diagonal) and p-values between all the samples. Note that all the male1 samples are statistically indistinguishable from each other. This is particularly apparent between the two aggregated male samples taken a week apart, despite displaying a relative lack of sequence overlap at the nucleotide level. All the male 1 samples are statistically distinguishable (p<0.05) from the second male sample and the female sample, indicating that the physico-chemical properties of these samples from different individuals are distinct.

### Generating P-values without Vβ Aggregation

It is theoretically possible that the significant ANOSIM statistics generated for samples between different individuals may have arisen from the aggregation of Kidera Factors with Vβ regions if there were preferential associations of particular CDR3 regions with different Vβ genes. To check this was not the case, we undertook ANOSIM tests using CDR3 Kidera Factor vectors from different individuals that were not aggregated with the Vβ regions. This procedure was implemented by randomly selecting two subsamples of one hundred CDR3 vectors from two individuals from their unique set of transformed CDR3 sequences. The ANOSIM statistic and subsequent P-value was then calculated between these two subsamples. This was repeated 100 times in a bootstrap sampling procedure that generated a distribution of the P-value for samples from the two given individuals.

**Figure 6:**
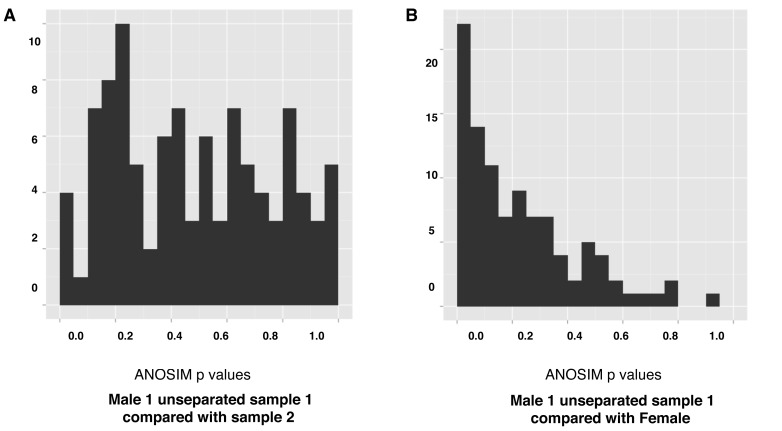
Distribution of the p-values in pairwise samples. The distribution of the p-values after bootstrapping non aggregated subsamples from both male1 unseparated samples (Figure 6A) and the male1 unseparated sample1 and female samples (Figure 6B). The relative uniform frequency of the p-values from 0 to 1 in (A) suggests that significant p-values would occur only by chance in these subsamples. This contrasts with the high number of significant p-values (p<0.05) in the distributions between the male1 and female samples (B).

This is a very computationally expensive analysis, which meant that the analysis could only be performed with a limited sample size (100 CDR3 vectors from each individual). Nevertheless, the results confirmed the findings obtained with the aggregated samples shown in Figure 5, with a random distribution of the P-values between 0.05–1 for the two unsegregated samples taken at different times from male 1 (Figure 6A), and a non-random distribution of the p-values for male 1 unsegregated sample 1 compared with the female sample (Figure 6B). This is denoted by the high frequency of significant p-value scores (p<0.05) in the R-statistic distribution shown in Figure 6B.

## Discussion

The TCR Vβ repertoires from three different individuals together with the naïve and memory repertoires from one individual were investigated using Kidera Factor representation of TCR CDR3 regions. By design, Kidera Factors capture uncorrelated physico-chemical properties of polypeptide chains and have been shown to delineate global protein structural properties encoded by distinct amino acid (and therefore gene) composition [19]. The Kidera Factor representation of CDR3 regions revealed considerable diversity, but less than that of the original TCR Vβ CDR3 DNA (and amino acid) sequencing [14] in the same samples (Table 1). This means that different CDR3 sequences can give rise to the same Kidera Factor vector. One interpretation of this observation is that different TCRs responding to the same antigen peptide may have similar 3D structural properties even if derived from distinct DNA sequences. This is an important notion if TCR diversity is to be measured only by DNA sequencing.

Notably, the distribution of CDR3 Kidera Factors obtained in different samples from the same individual could not be distinguished either by PCA or by MDS analysis even though there was only a small overlap (∼10%) in the corresponding CDR3 DNA sequences. This result suggests that relatively small sampling can potentially capture the population diversity within an individual, at least in terms of the TCR physicochemical (structural) properties. In this case, an individual’s TCRβ diversity as indicated by Kidera Factors may be somewhat smaller than is allowed by the theoretical range of potential CDR3 sequences produced by hyper-recombination during T cell development. There was also a similar distribution of CDR3 Kidera factors shown by PCA in naïve and memory cell populations from the same individual despite only about 1% overlap in CDR3 DNA sequences and 14% overlap in amino acid sequences. This is consistent with the memory cell compartment being a random sample of the naïve population. It is important to bear in mind however that the Kidera Factor diversity described here is of the TCRβ chain CDR3 segment only and does not give information about either contribution by residues in V or J regions and more importantly the TCRα/β dimer that binds to antigen/MHC. Interestingly, there appeared to be some aggregation of the memory cell Kidera Factors, which might be interpreted as restricted heterogeneity of the repertoire to particular infections. It would be of particular interest to look into this further with T cell populations taken during or just after an acute infection.

When comparing samples from different individuals (Figure 3), the distributions shown by PCA alone appeared to be slightly different. To investigate this further, the Kidera Factors were first aggregated with the Vβ genes they were associated with in the identified clonotypes and then subjected to MDS analysis. A clear difference between samples from different individuals was evident by this analysis but not samples from the same individual taken at different times or for naïve and memory samples from the same individual (Figure 4). Moreover, the ANOSIM statistic showed these differences were highly statistically significant (Figure 5, Table 2). Because the Kidera Factors were aggregated by Vβ gene, it was theoretically possible that the differences between individuals could be due to different Vβ gene usage resulting from selection of the TCR on different sets of MHC self peptides. To investigate this possibility, repeated samples of smaller numbers of non-aggregated Kidera Factors from different donors and in different samples from the same donor were subject to ANOSIM analysis (Figure 6). The results from these analyses supported the conclusion from the Vβ aggregated results showing that samples from different individuals could be distinguished whereas samples taken at different times or naïve and memory T-cell samples from the same individual could not. The biological significance of this finding is not quite clear at this stage. If the structural properties of the Vβ chain identified by the Kidera factors are specific for antigen binding, the similar distribution in naïve and memory T-cell and in samples taken at different times from the same individual may suggest a smaller antigen specific repertoire than indicated by the sequences alone. Alternatively, the “structural properties” identified by the Kidera Factors may reflect Vβ chain binding to different MHC types rather than antigen.

It is important to note that these results were obtained despite considerable information compression. The CDR3 amino acid sequences were turned into a ten dimensional vector representing the averaged Kidera Factors of the amino acids composing the sequence so that information inferable from the order of the amino acids was lost in the process. Nevertheless, Kidera Factor representation of the CDR3 conveys information that is related to protein structure and gives information about the TCR repertoire that is not evident from DNA or amino acid sequences alone [19], [20]. This could have important implications for relating the TCR repertoire to antigen recognition and in the clinical arena such as ageing, autoimmune disease and immune reconstitution in HIV after anti-retroviral treatment and following stem cell transplantation.

## Supporting Information

Figure S1
**shows how permuting the sample labels and recalculating the R-statistic generate the null distribution for the ANOSIM test.** Permuting the labels produces an alternative plausible distribution of the aggregate labels if, under the null hypothesis, the samples cannot be distinguished and are effectively replicates from the same sample. To obtain a p-value, the R statistic must be compared with a null distribution. As the ANOSIM test is a non-parametric test, this null distribution must be computed. The distribution of rank similarities under the null hypothesis implies that the samples containing the CDR3 Kidera Factors are indistinguishable from each other. Taking the null hypothesis to be true, each CDR3 Kidera Factors is effectively just a replicate from a single sample. Randomly re-assigning the sample labels (i.e. either “sample 1” or “sample 2”) among the CDR3 Kidera Factor vector represents an alternative permutation of the sample labelling of the CDR3 Kidera Factors. If the samples are indeed indistinguishable, this random reassignment can be used to generate a null distribution by repeatedly shuffling and re-assigning the sample labels pertaining to each aggregated gene (Figure S1). This maintains the rank similarity between pairs of CDR3 Kidera Factors but reassigns each aggregate at random between the two samples. An R-score is computed by using the same formula above for each random reshuffle. Summating these R-scores creates the null distribution. A p-value for the original R-statistic can now be obtained by evaluating the proportion of reshuffles for which the permuted R-statistics exceeds or equals the actual R-statistic. Thus instead of relying on the value of R, we can qualify the difference between samples on the p-value, where the smaller it is, the lower the chance that the actual permutations of labels has come from the null distribution and hence the more likely the samples are to be different. In practice, given the number of possible combinations (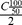) for two samples, each with 50 aggregated Kidera Factor data points, a subsample of these combinations can be generated to serve as the null distribution. Sampling 1,000 permutations by randomly assigning the sample labels to each gene in the similarity rank matrix generates a null distribution of sufficient size to be able to accept or reject the null hypothesis (Figure S1 for schematic).(PDF)

Table S1 **Gives the 10 dimensional Kidera Factor descriptions with values for alanine as an example of all twenty amino acids taken from reference 18.**
(DOCX)
