# Supplementary material for: Revealing Individual Signatures of Human T Cell CDR3 Sequence Repertoires with Kidera Factors
Source: PLoS One. 2014 Jan 29;9(1):e86986. doi: 10.1371/journal.pone.0086986 (PMC3906109; doi:10.1371/journal.pone.0086986)
Supplement: Figure S1 — shows how permuting the sample labels and recalculating the R-statistic generate the null distribution for the ANOSIM test. Permuting the labels produces an alternative plausible distribution of the aggregate labels if, under the null hypothesis, the samples cannot be distinguished and are effectively replicates from the same sample. To obtain a p-value, the R statistic must be compared with a null distribution. As the ANOSIM test is a non-parametric test, this null distribution must be computed. The distribution of rank similarities under the null hypothesis implies that the samples containing the CDR3 Kidera Factors are indistinguishable from each other. Taking the null hypothesis to be true, each CDR3 Kidera Factors is effectively just a replicate from a single sample. Randomly re-assigning the sample labels (i.e. either “sample 1” or “sample 2”) among the CDR3 Kidera Factor vector represents an alternative permutation of the sample labelling of the CDR3 Kidera Factors. If the samples are indeed indistinguishable, this random reassignment can be used to generate a null distribution by repeatedly shuffling and re-assigning the sample labels pertaining to each aggregated gene (Figure S1). This maintains the rank similarity between pairs of CDR3 Kidera Factors but reassigns each aggregate at random between the two samples. An R-score is computed by using the same formula above for each random reshuffle. Summating these R-scores creates the null distribution. A p-value for the original R-statistic can now be obtained by evaluating the proportion of reshuffles for which the permuted R-statistics exceeds or equals the actual R-statistic. Thus instead of relying on the value of R, we can qualify the difference between samples on the p-value, where the smaller it is, the lower the chance that the actual permutations of labels has come from the null distribution and hence the more likely the samples are to be different. In practice, given the number of possible combinatio [file pone.0086986.s001.pdf]

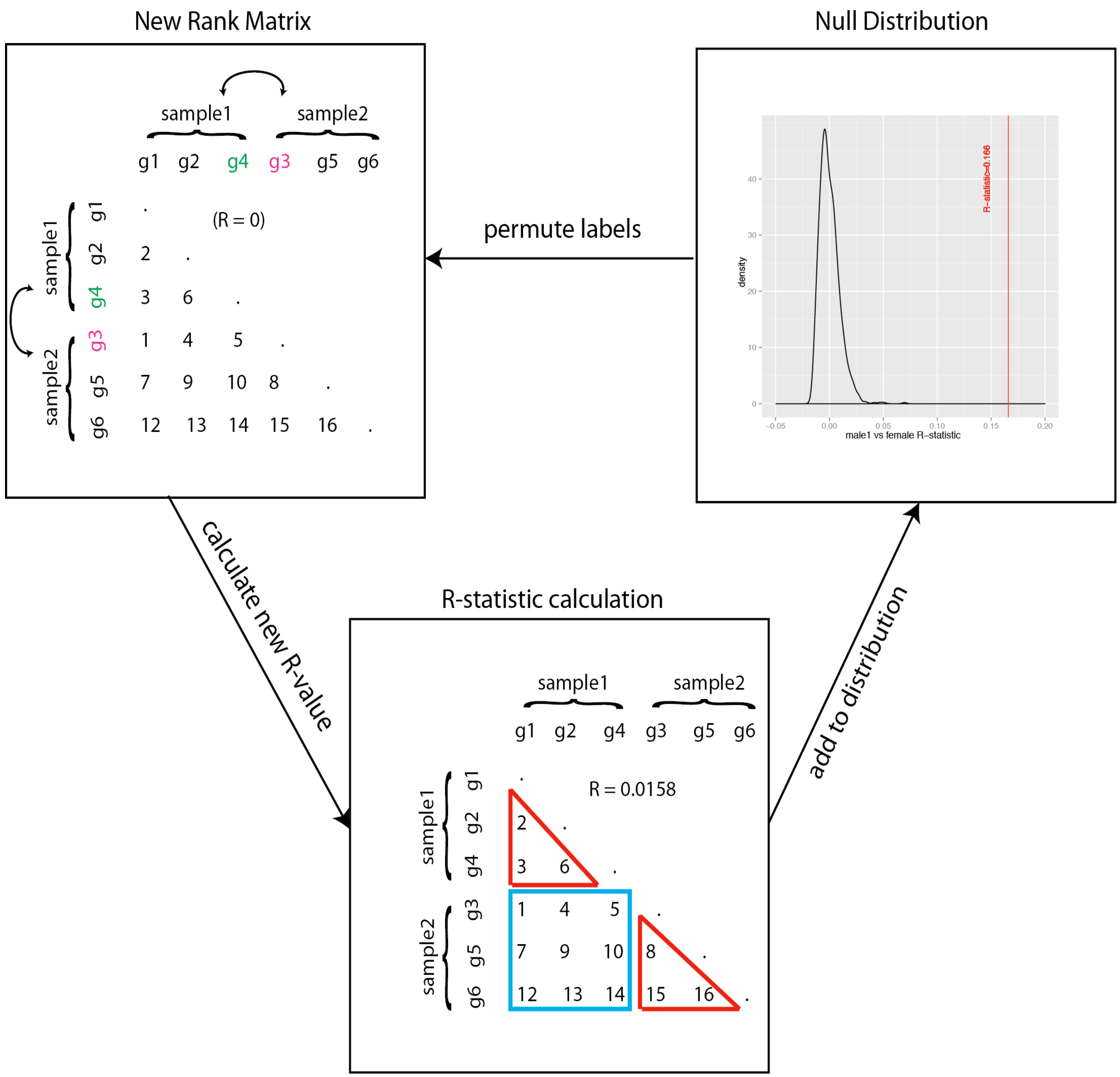
New Rank Matrix
sample1
sample2
g1 g2 g4 g3 g5 g6
(R = 0)
permute labels
Null Distribution
R–statistic=0.166
density
male1 vs female R–statistic
calculate new R-value
R-statistic calculation
R = 0.0158
add to distribution
