# Supplementary material for: Revealing Individual Signatures of Human T Cell CDR3 Sequence Repertoires with Kidera Factors
Source: PLoS One. 2014 Jan 29;9(1):e86986. doi: 10.1371/journal.pone.0086986 (PMC3906109; doi:10.1371/journal.pone.0086986)
Supplement: Table S1 — Gives the 10 dimensional Kidera Factor descriptions with values for alanine as an example of all twenty amino acids taken from reference 18. (DOCX) [file pone.0086986.s002.docx]

## Table S1 : Description of Kidera Factors

| **Kidera Factor No** | **Description** | **Example Score for Ala** |
| --- | --- | --- |
| 1 | Alpha-helix/bend preference | -1.56 |
| 2 | Side-chain size | -1.67 |
| 3 | Extended structure preference | -0.97 |
| 4 | Hydrophobicity | -0.27 |
| 5 | Double-bend preference | -0.93 |
| 6 | Amino acid composition | -0.78 |
| 7 | Flat extended preference | -0.2 |
| 8 | Occurrence in an α-region | -0.08 |
| 9 | pK | 0.21 |
| 10 | Surrounding hydrophobicity | -0.48 |
